# Remote Sensing and Landcover in Ring‐Necked Pheasant Research: A Review of Data Sources and Scales

**DOI:** 10.1002/ece3.71576

**Published:** 2025-06-21

**Authors:** Megan Baldissara, Allison Barg, Andrew Little, Zhenghong Tang, Brian Wardlow, Daniel R. Uden

**Affiliations:** ^1^ Applied Wildlife Ecology and Spatial Movement Lab, School of Natural Resources University of Nebraska‐Lincoln Lincoln Nebraska USA; ^2^ Community and Regional Planning Program University of Nebraska‐Lincoln Lincoln Nebraska USA; ^3^ Center for Advanced Land Management Information Technologies, School of Natural Resources, Institute of Agriculture and Natural Resources University of Nebraska‐Lincoln Lincoln Nebraska USA; ^4^ Department of Agronomy and Horticulture, Center for Resilience in Agricultural Working Landscapes, School of Natural Resources University of Nebraska‐Lincoln Lincoln Nebraska USA

**Keywords:** extent, grain, habitat, remote sensing, wildlife

## Abstract

Documenting wildlife–habitat relationships at multiple scales is essential for conservation. Remote sensing datasets and their derivatives (e.g., landcover data) enable efficient multi‐scale assessment of ring‐necked pheasant ( *Phasianus colchicus* ) habitat, albeit with trade‐offs among their thematic, spatial, temporal, and/or spectral grains and extents. For example, the National Agriculture Imagery Program provides fine spatial but coarse spectral grain imagery, both important for identifying pheasant habitats. Spatial technologies and datasets relevant to pheasant research are advancing, yet the information on the data sources utilized in research to date is limited. Remote sensing and landcover datasets surveys in pheasant research could help fill information gaps in pheasant–habitat relationships. In this systematic review, we filtered 1110 peer‐reviewed pheasant habitat studies to 65 from the Central United States. Temporal trends were tested in the broad use of remote sensing and the selection of remote sensing platforms and data types. Of the selected studies, 26 used remote sensing or landcover data, which were classified by the thematic, spatial, temporal, and spectral grains and extents. Remote sensing and landcover data products increased over time, particularly satellite‐based landcover products with relatively coarse thematic resolutions (e.g., crops and grassland), moderate spatial grains (e.g., 30‐m), and spatial extents (e.g., smaller than the average US county). Remote sensing photography/imagery with multispectral sensors and coarse spectral resolution (e.g., three bands with 100 nm width) was also prominent but remained constant over time. We found no evidence of research with remote sensing or landcover data at multiple temporal grains and extents. Several studies lacked scale reporting, potentially limiting our inference. Scale transparency is important due to species selecting their habitat at multiple scales, making findings scale‐dependent. Effective conservation requires scale‐appropriate strategies. As remote sensing advances, opportunities for ring‐necked pheasant habitat multi‐scale assessment that fill remaining pheasant–habitat relationships knowledge gaps and support management decisions will increase.

## Graphical abstract

While remote sensing datasets provide valuable tools for assessing ring‐necked pheasant habitat, there is a notable lack of information on the data sources used in existing research. In a systematic review, we found that only 40% of studies utilized remote sensing or land cover data, primarily using satellite‐based products with coarse resolutions; however, many studies failed to report the scales used, hindering our understanding of pheasant habitat at multiple temporal scales. As remote sensing technology advances, addressing scale biases will be essential for effectively managing pheasant habitats.

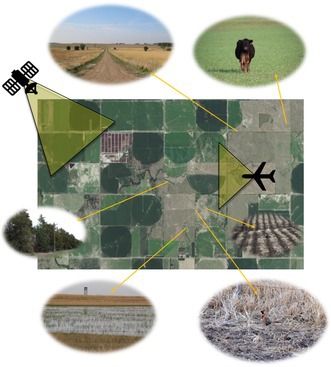

## 1 Introduction

Understanding of hierarchical wildlife–habitat relationships emerges from multi‐scale research. Ignoring scale dependencies can lead to misleading ecological conclusions and misplaced conservation management (Johnson 1980; Wiens 1989; Levin 1992; Godvik et al. 2009; Mayor et al. 2009). Ecological scale refers to spatial and/or temporal dimensions described and analyzed in a study. Scale is characterized by the grain, which is the smallest discernible unit measured in a study (resolution) and the extent, which is the total area or time under investigation. A change in either the grain or the extent will alter the scale of the study (Turner et al. 2001; McDermid et al. 2009; Maxie et al. 2010). Multi‐scale habitat selection describes habitat selection in multiple spatial and temporal scales, from local, short time decisions to broad geographic patterns during long periods (Mayor et al. 2009; McGarigal et al. 2016). Habitat selection is influenced by factors like predation risk, resource accessibility, and reproduction, all of which can vary with scale. For example, the little bustard ( *Tetrax tetrax* ) prefers fallows and pastures at broad spatial extents but selects specific plant structures at finer spatial extents (Traba et al. 2022). Multiscale habitat research helps ensure the identification of critical habitat selection factors. Several reviews have addressed multiscale wildlife habitat relationships, however, without assessing the use of remote sensing (Gaillard et al. 2010; McGarigal et al. 2016; Tellería 2016). Remote sensing is a revolutionary tool in wildlife ecology, able to provide accessible habitat data at multiple ecological scales (Homer et al. 1993; Viña et al. 2008; Vogeler and Cohen 2016; Marvin et al. 2016).

**FIGURE 1:**
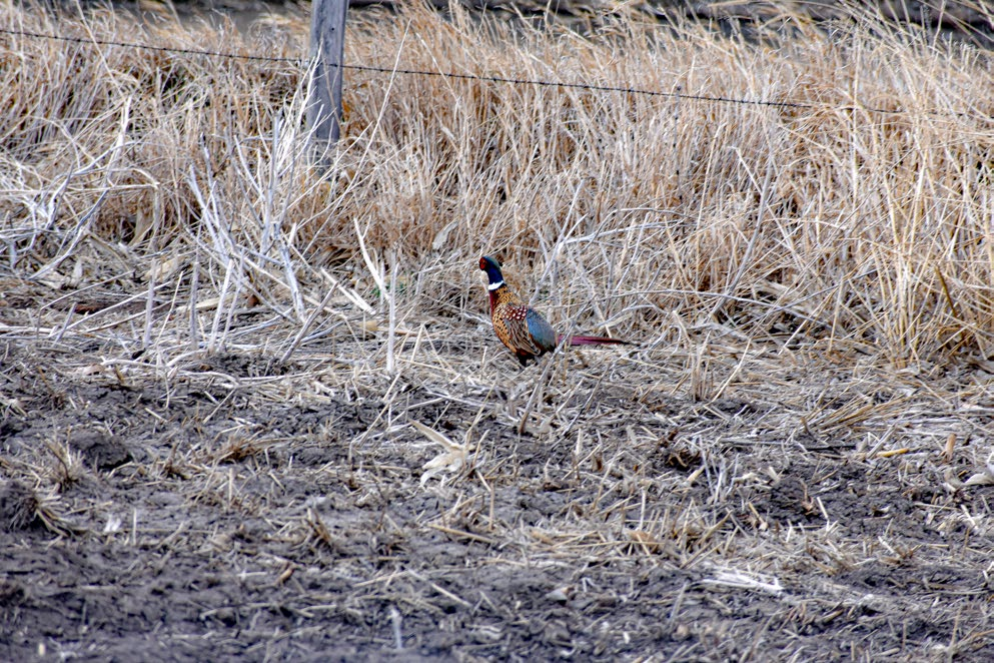
Ring‐necked Pheasant (*Phasianus colchicus*) in Rural Nebraska. Photo credit: Aaron Keanini.

Remote sensing can be used for multi‐scale pattern detection; however, it is often utilized at coarse grains. Field sampling of variables like vegetation height and species composition is often utilized at fine grains. In this manuscript, scale refers to pre‐modeling scaling, before it is modified for modeling purposes (Fritsch et al. 2020). Remote sensing has successfully researched wildlife habitat at multiple scales, providing important insights for conservation efforts (Homer et al. 1993; Viña et al. 2008; Vogeler and Cohen 2016). For instance, the Landcover Map 2000, derived from SPOT 4 satellite imagery, was used to discover the preference of ground‐nesting birds for homogenous habitats at a fine spatial extent but not at broad ones (Pickett and Siriwardena 2011). MODIS and Sentinel‐2–derived landcover were used to discover the European Nightjar's ( *Caprimulgus europaeus* ) preference for grassland availability at a fine spatial extent and diverse landscape at broad extents (Lathouwers et al. 2023). Such findings showcase the utility of remote sensing in multiscale habitat research and management. Yet remote sensing is not prominent in the research of the habitat of the ring‐necked pheasant ( *Phasianus colchicus* Figure 1), a culturally, economically, and ecologically important species across central North America. Pheasants were introduced successfully in North America in 1881 for hunting purposes, becoming widespread and naturalized. Pheasants are important indicators of ecosystem health in environments heavily disturbed by humans, and understanding their habitat requirements and survival informs management practices that affect other species in such environments (Prince et al. 1988; Haroldson et al. 2006; BirdLife International 2016; Pauly et al. 2018; Augustine et al. 2021). Pheasant habitat relationships have been researched for decades; however, at a relatively narrow ranges of scales, limiting ecological understanding and management outcomes (Baxter and Wolfe 1973; Rodgers 1973; Keyser III 1986; Jorgensen et al. 2014).

Pheasant habitat includes grasslands, cropland, woodland, and wetlands (Berner 1988; Robertson et al. 1993; Taylor et al. 2018; Harsh et al. 2022); however, many remote sensing and landcover pheasant studies focus on certain habitat classes and/or represent them at coarse thematic grains, such as grassland or forest (e.g., Schindler et al. 2020; Emmet et al. 2023; Shirley and Janke 2023). Thematic grains are levels of landcover classification that correspond with general‐to‐specific habitat classifications (e.g., from the general class of cropland to maize [ *Zea mays* ] or other specific crop types; Tuanmu and Jetz 2015; Pelorosso et al. 2021; Price et al. 2023). Such classification may not capture habitat heterogeneity, from the perspectives of different species, such as the differences in grassland structure at different times since disturbance which can be important for pheasants (Van Andel and Van den Bergh 1987; Warner et al. 2000). Pheasant habitat selection can be described in terms of spatial and temporal scale. Spatially, pheasant's home range varies from 5 to 300 ha and its geographic range covers a substantial portion of the North American continent, making spatial extent an important factor in study design. Thematic and spatial grain interact, for example, detecting early successional grasslands depends on both spatial resolution and classification detail. Pheasant habitat needs vary during different life stages: nesting, brood rearing, and overwintering. The temporal grain (observation frequency) and extent (study time frame) of pheasant and habitat observations are important in detecting temporal pheasant habitat selections (Whiteside and Guthery 1983; Hill and Ridley 1987; Perkins et al. 1997; BirdLife International 2016). The most appropriate spatial and temporal grains depend on pheasant ecological needs and must be able to distinguish habitat features during different life stages. For instance, during nesting (March–April), dense grassland is needed for cover, while during brood rearing (May–June), less dense grassland is required for facilitated movement and foraging. During foraging periods (July–August and September–November) and winter (December–February), pheasants rely on a mix of grassland, cropland, wetlands, and woodland types for food and shelter (Riley et al. 1998; Clark et al. 1999; Rodgers 2002; Leif 2005; Amirkhiz et al. 2023; Barg et al. 2025). Despite having the potential to model habitat needs across multiple spatial–temporal scales, the use of remote sensing is not yet commonplace in pheasant research (Théau 2008; Campbell and Wynne 2011b; Gibson and Power 2013a, 2013b; Emmet et al. 2023; Shirley and Janke 2023).

Although several pheasant studies have adopted multi‐scale modeling frameworks, it is generally multiple spatial scales, rather than temporal or thematic scales, that were compared (Harsh et al. 2022; Shirley and Janke 2023). For example, Shirley and Janke (2023) investigated pheasant habitat selection at three spatial scales during one nesting season. At all spatial scales, crops were not important for pheasants; however, they could have been at different life stages and times of year, such as brood rearing (Godar et al. 2023). This highlights the importance of temporal scale when assessing pheasant habitat. Amirkhiz et al. (2023) are one of the few pheasant studies that incorporate ecologically relevant spatial and temporal scales using remote sensing techniques. They found that pheasants prefer intermediate levels of grassland, especially at the local spatial extent; however, other variable importance varied yearly depending on weather and climatic conditions. Scale selection should be guided by pheasant ecology, research focus, and data availability (Barg et al. 2025). Remote sensing is often utilized across broad spatial extents with a broad research focus due to its ability to increase efficiency in monitoring; however, it can also be effective at smaller extents. In some cases, limitations in the extent over which pheasant field data (e.g., crow counts) are available may constrain the use of remotely sensed habitat data. It is unclear whether underutilization of remote sensing in pheasant research is due to technology and data not being used to their full potential or an inability to identify pheasant habitat features at relevant scales. Identifying the abilities and limitations of remote sensing in assessing pheasant habitat has the potential to enhance understanding of pheasant habitat relationships, guide research, and inform management (Baxter and Wolfe 1973; Rodgers 1973; Keyser III 1986; Jorgensen et al. 2014).

Remote sensing and landcover data collected at ecologically relevant scales have the greatest potential to contribute to understanding of pheasant–habitat relationships. The use of landcover data tends to be more common in pheasant research than vegetation indices like the normalized vegetation index (NDVI) derived from satellite imagery (Campbell and Wynne 2011a; Hansen and Loveland 2012; Shahbazi et al. 2014). However, tradeoffs among the spatial, temporal, and thematic extents and grains of data can pose challenges. For example, a dataset might offer high spatial resolution (i.e., fine grain) information, but only over a small extent. Or a dataset might provide information over a large geographic extent, but at low spatial resolution (i.e., coarse‐grain; Wiens 1989; Kushwaha and Roy 2002; McDermid et al. 2009; Vogeler and Cohen 2016). Even if spatially high‐resolution data is available at a large spatial extent, it may only be produced infrequently (i.e., at a coarse temporal resolution) or may be incapable of differentiating between ecologically meaningful habitat features, such as different successional stages in grassland or woodland ecosystems (i.e., coarse thematic resolution in landcover data). Scale‐based tradeoffs in remote sensing and landcover data emerge from several sources. Grain‐extent tradeoffs vary with remote sensing platforms, such as drones, aircraft, and satellites. Among these, drones capture information at the finest spatial grain and smallest spatial extents, aircraft provide information at coarser spatial grains and larger spatial extents, and satellites provide data at the coarsest spatial grains and largest spatial extents. Finer spatial grain in remotely sensed data supports the more detailed assessment of habitat features (Wulder et al. 2004; Campbell and Wynne 2011b; Johnson 2013; Palchowdhuri et al. 2018). Different remote sensing platforms also support sensors with varying spectral grain (the number and width of spectral bands), which is important for habitat and landcover classification. The finer the spectral resolution (smaller increments of spectral quantification across a greater number of bands), the greater the capacity to classify specific habitats relevant to pheasants at finer thematic grains (e.g., early successional and biodiverse grassland; Marcus 2002; Legleiter 2003; Bradter et al. 2020; Barg et al. 2025). Information from remote sensing and landcover can inform decisions at multiple scales relevant to management, not just those with the greatest logistical or economic relevance (Erasmus et al. 1999; Fjeldså 2007; Lechner et al. 2015). Our ability to overcome constraints in the use of remote sensing and landcover data in pheasant research and management is hampered by knowledge gaps about multi‐scale pheasant–habitat relationships.

Spatial technologies and datasets relevant to pheasant–habitat relationship studies are advancing. Yet there is a lack of information on the data sources and spatial, temporal, thematic, and spectral scales utilized in research to date. Such informational gaps limit the ability to determine how remote sensing data are applied to pheasant research, potentially leading to this technology overlooking important ecological patterns. In this comprehensive review of the use of remote sensing and landcover data in peer‐reviewed pheasant habitat studies in the central United States, we (1) categorize pheasant habitat studies based on the use of remote sensing (including remote sensing indices) and landcover data, data collection platforms, data types, and data scales (thematic, spatial, temporal, and spectral grains) and extents. The thematic grain is the level of detail of discrete land use/land cover typologies with defined boundaries. A finer thematic grain differentiates subcategories, like specific woodland types within a broad forest category. Spatial grain is defined as the smallest discernible unit of observation, spatial extent is the overall area under evaluation, temporal grain is the smallest discernible unit of time, and temporal extent is the total length of time under consideration. The spectral grain refers to the ability of sensors to distinguish between wavelengths. It was measured by the number of bands used in the study and the wavelength difference width range within the bands (Campbell and Wynne 2011b; Gibson and Power 2013a, 2013b; Turner et al. 2001); (2) test for temporal trends in dataset characteristics to determine how the use of different data sources, platforms, and methodologies has evolved over time; and (3) evaluate remote sensing products suitable for pheasant habitat assessments and encourage future technology development for the benefit of pheasant habitat research at different scales. The scope of the review is the current use of remote sensing at different scales in pheasant research in central United States from the earliest available records to 2023. Documentation and evaluation of the spatial, temporal, spectral, and thematic scales of data used in research to date is useful for guiding research to close remaining knowledge gaps in pheasant–habitat relationships and inform selection of datasets and scales for maximum management utility. This study directly addresses important concerns related to pheasant conservation and habitat management by concentrating on central United States. Understanding how pheasants interact with agricultural landscapes and grassland ecosystems can help inform more effective management strategies and prioritize conservation efforts in regions where they are most needed (Reeves et al. 2021; BirdLife International 2016; Augustine et al. 2021; Barnes et al. 2025).

## 2 Material and Methods

### 2.1 Study Area

**FIGURE 2:**
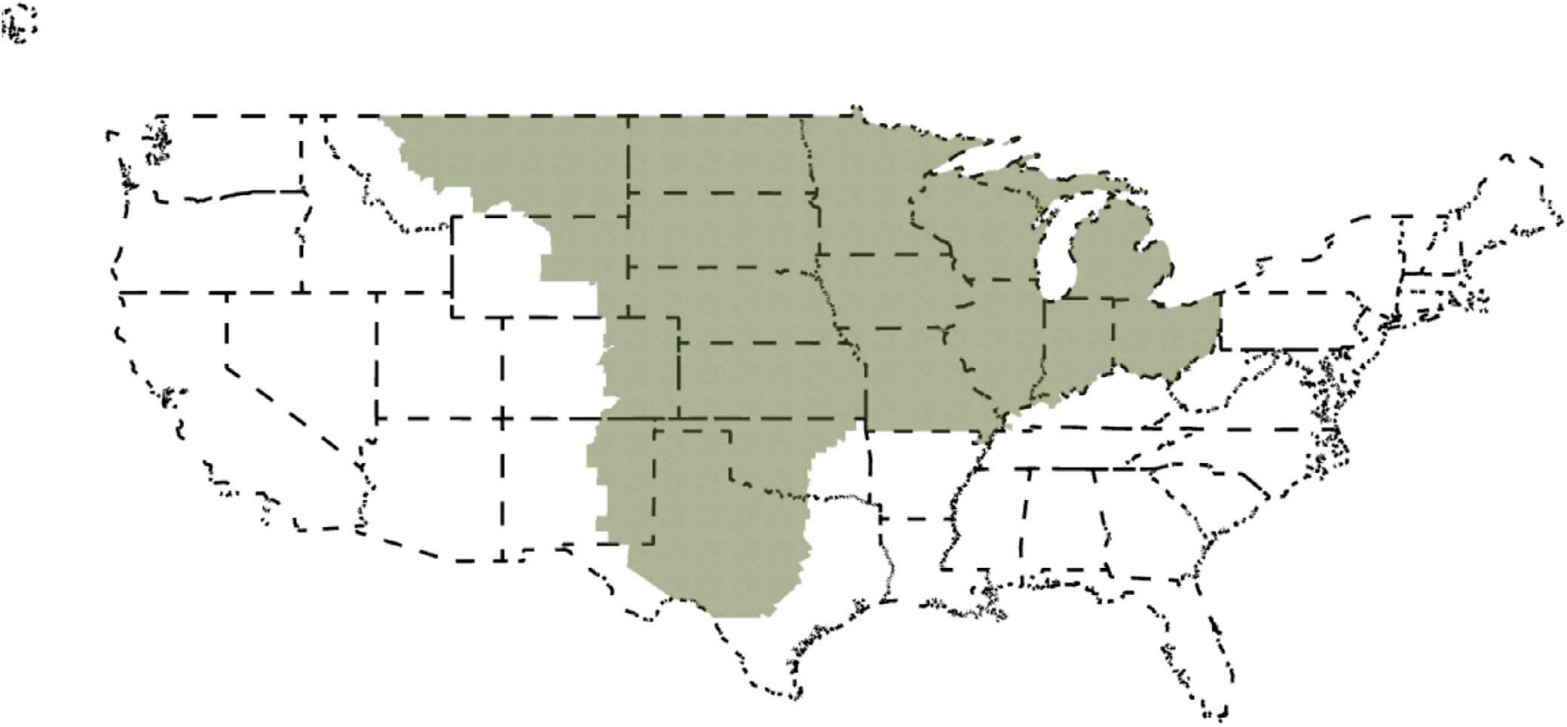
Geographical inclusion criteria of pheasant (*Phasianus colchicus*) studies in the review (indicated in green). The map was created with ESRI (2024) and with U.S. Census Bureau (2020) data.

The review systematically collected all available papers that qualitatively or quantitatively researched pheasant habitats across the Great Plains, Midwest, and the Corn Belt regions of the United States. We used the Great Plains regional delineation of Lavin et al. (2011), the Midwest delineation of National Geographic (2024), and the Corn Belt delineation of Green et al. (2018; Figure 2). These regions were selected to collectively capture central North America and the most concentration of pheasant populations. This area contains critical habitats for pheasants in mixtures of croplands, grasslands, woodlands, and wetlands, which are essential for different stages of the species' life cycle. The region, however, is facing significant land use changes due to agricultural intensification, urbanization, and climate change (Reeves et al. 2021; BirdLife International 2016; Augustine et al. 2021; Crimmins et al. 2023; Barnes et al. 2025).

### 2.2 Research Selection and Classification Criteria

**FIGURE 3:**
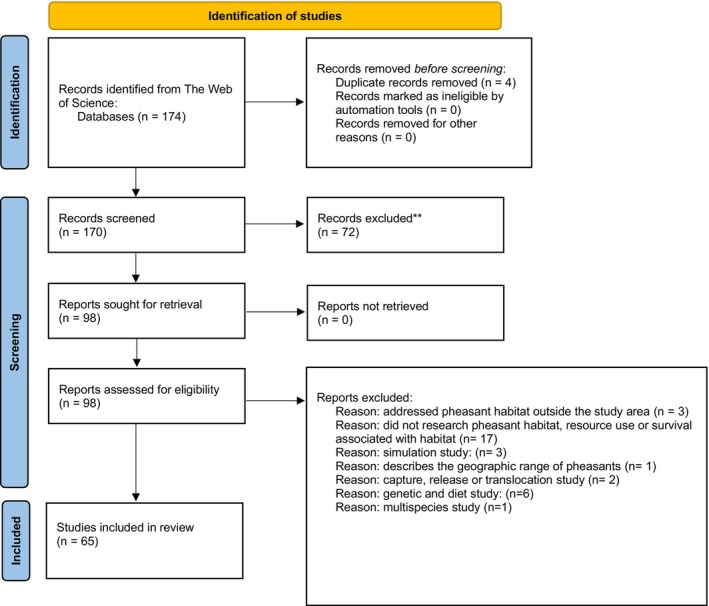
Identification, screening, and inclusion of the literature selected for analysis for this review. This work is licensed under a Creative Commons Attribution 4.0 International License (CC BY 4.0). For more details or to view the license, visit https://creativecommons.org/licenses/by/4.0/.

Two authors of this paper independently collected and filtered articles following the same protocols. The search was performed in May 2024. The results from each author were then compared to check that the process was performed correctly. The review followed the PRISMA guidelines for ecology and evolution (O'Dea et al. 2021; Page et al. 2021), utilizing all databases and collections in the Web of Science. The criteria for developing the search query are listed in Table 1. The final search query returned papers with titles or abstracts containing keywords related to pheasants and their habitats. The search initially returned 1100 papers, which we filtered to 174 peer‐reviewed articles based in the United States or Canada. These papers were further filtered according to Figure 3 and Table 2 based on species focus, study type, study area, research and data scope, study design, and whether they involved translocation, genetic, diet, and multiple species.

**TABLE 1:** Research criteria for ring‐necked pheasant ( *Phasianus colchicus* ) habitat papers showing the series of seven searches used to generate the initial set of papers.

Search number	Search location	Keywords	Search number combination
1	Title	pheasant OR “ *Phasianus colchicus* ”	
2	Abstract		
3	Title	habitat OR mesohabitat OR macrohabitat OR microhabitat OR “food plots” OR agriculture OR cover‐type OR “nest site” OR “land use” OR “resource selection” OR “resource use” OR “cover preferences”	
4	Abstract		
5			#1 OR #2
6			#3 OR #4
7			#5 AND #6

**TABLE 2:** Manual exclusion criteria of pheasant papers.

Base of paper exclusion	Excluded papers	Reason for exclusion
Species focus	Paper did not focus on the ring‐necked pheasant ( *Phasianus colchicus* )	The review is specific on the ring‐necked pheasant ( *Phasianus colchicus* )
Study type	Paper was a non‐original primary peer‐reviewed research article	Ensures inclusion of only high‐quality research
Study area	Paper addressed pheasant habitat outside the study area (Great Plains, Midwest, and Corn Belt core area; Figure 2). Studies that only covered a portion of the study area were included. If no location information was available, the study was considered outside the study area	Ensures studies are within the study area
Research Scope	Paper did not research pheasant habitat, resource use, or survival associated with habitat	Ensures inclusion of only habitat‐related research
Data source	Paper developed models with data from other studies	Avoids replication from other studies
Study design	Paper was based on a simulation or described the geographic range of pheasants	Ensures inclusion of only habitat‐related research from real data
Translocation	Paper focused on capture, release or translocation. Studies that used a mix of wild and captive/translocated pheasants were excluded if the majority were captive or translocated. If a study had both distinct captive or translocated and wild pheasants in a wild setting, only results from the wild pheasants were regarded	Ensures relevance to wild pheasant populations and not captive ones which may display different habitat selection processes
Genetic and diet studies	Paper did not provide information about pheasant habitat but only about pheasant genetics and diet	Ensures inclusion of only habitat‐related research
Multispecies studies	Paper did not focus on pheasant habitat but instead focused on biodiversity or richness metrics	Ensures focus on the ring‐necked pheasant ( *Phasianus colchicus* )

Only papers that used remote sensing, landcover, or vegetation indices were utilized in the scale classification. Papers using other variables such as land surface temperature or soil moisture were not considered, to maintain a focus on pheasant habitat and its characteristics. To address the time lag between the time the research was conducted and the publication year, which can inflate or deflate temporal findings, all papers were considered, regardless of when they were published. Remote sensing originates over a century ago with early technology using aerial photography from planes and even passenger pigeons. The inclusion of older studies illustrates the influence of fundamental methods in modern approaches to pheasant habitat research, especially studies from the mid‐20th century onward. Such early studies provide historical context relevant to understanding long‐term trends and methodology advancements (Gibson and Power 2013a, 2013b). This process yielded a final set of 65 articles.

We classified the 65 studies according to information found in their Introduction, Study Area, and Methods sections. Within the Methods sections of the 65 studies, we searched for keywords such as aerial, imagery, photography, remote sensing, landcover, and land use to determine whether the study used remote sensing and/or landcover data. Mixtures of remote sensing and landcover data with additional data, such as field observations and management records, were classified as utilizing these data types. For the studies that did use remote sensing and/or landcover, we noted whether or not ground‐truthing was employed. Studies that used remote sensing or landcover were categorized based on the remote sensing data collection platform (aircraft, satellite, or unknown) and the data type (photographs, landcover, indices, or unknown). The unknown categories for each platform, data type, and scales were added to deal with missing information, report study quality, and report their occurrence.

From the 65 studies, we calculated the percentage of articles researching pheasant habitat using remotely sensed or landcover data products as a data source. If the study area or remote sensing use was not clearly stated, we regarded those studies as outside the specific study area and as not using remote sensing. For example, if a paper stated that their study area was in a region that overlaps our study area but does not specify where specifically in the region the study was conducted, it was counted as outside our study area. This ensured that the reviewed papers were conducted in our study area and used remote sensing. We also determined the proportion of different remote sensing platforms and data types used in these articles. Percentages were calculated by dividing the usage of each platform or data type by the total usage of remote sensing products across all studies. We also determined the proportional use of different remote sensing datasets. This was calculated by dividing the times each remote sensing dataset was used by the total number of times all the remote sensing datasets were used. To understand the effect of the missing information described by the unknown categories, percentages were also calculated excluding such categories. All percentages were rounded to ensure a collective sum of 100%. The presence of multiple remote sensing products with different platforms and data types in the same studies was accounted for when calculating percentages.

### 2.3 Temporal Trends

We conducted the Mann–Kendall tau trend test in R (version 4.4.0) with the “Kendall” package accounting for ties to examine changes in pheasant habitat publication types over time. Ties occur when numerous values are identical in a dataset (McLeod 2022; R core Team 2024). The test was employed to detect whether remote sensing use in the pheasant habitat literature increased or decreased over time. This analysis was also performed to test long‐term temporal patterns in pheasant habitat publications that utilize remote sensing, providing insights on the evolution of research methodologies used over time. This is a non‐parametric test that identifies positive or negative trends in a time series by comparing each point to the subsequent one. This test is well suited for non‐normal data, such as the one we had, and can handle outliers well, being an optimal choice for assessing temporal trends in pheasant studies. The outputs of the test are Kendall's tau coefficient indicating the strength and direction of the relationship and the *p*‐value indicating the significance of the relationship (Kendall 1945; Sen 1968; McLeod 2022). The tidyr package was used to structure and organize the data, while the ggplot2 and patchwork packages were used for plot visualizations (Wickham 2016; Pedersen 2024; Wickham et al. 2024).

### 2.4 Thematic, Spatial, and Temporal Scale

Papers that used remote sensing or landcover data (*N* = 26) were classified based on the thematic, temporal, spatial, and spectral grain, and temporal and spatial extent with methodologies developed by the authors. Spectral grain (ability of sensors to distinguish between wavelengths) excludes landcover and indices usage in remote sensing. We calculated the percentages of papers using different thematic and spectral resolutions and spatial and temporal grains and extents. The thematic categories are hierarchically listed in Table 3, from Level 1 (most general) to Level 5 (most specific in categorization). The National Land Cover Dataset (NLCD) classification system was the base for Levels 3 and 4 of the classification, which is a modified version of the Anderson Land Cover Classification System (Anderson et al. 1976; U.S. Geological Survey 2019). The finer levels of classification are integrated from other standard thematic schemes. The Conservation Reserve Program (CRP) land classification was integrated into level 4 from the Hierarchical All Bird System (HABS) habitat classification that identifies bird habitats at multiple scales (McLachlan et al. 2007). Only shrubs, CRP practices, cultivated crops, herbaceous wetlands, and open water were described by pheasant studies in more detail for level 5 categorization. These levels were categorized according to the USDA CRP practice library, the Cropland Data Layer (CDL) classification, and the Stewart & Kantrud Classification System. The latter is a widely used method for wetland and water systems classification (Stewart and Kantrud 1971; Farm Service Agency 2024; National Agricultural Statistics Service 2024). No standardized classification was used for shrubs in Level 5 due to only one paper characterizing shrubs at the species level. Level 2 group classes include categories that are closely related (share similar landcover characteristics) in Level 3 in the NLCD classification, except for wetland and water, as they have in common the presence of an aquatic environment. Level 1 provides a general classification of all the classes previously described.

**TABLE 3:** Hierarchical organization of land use and land cover classes, with Level 1 being the most general (i.e., coarse thematic resolution) and Level 5 being the most specific (i.e., fine thematic resolution).

Level 1	Level 2	Level 3	Level 4	Level 5
Terrestrial and aquatic environments	Forest and shrub/scrub	Forest	Deciduous forest	
			Evergreen forest	
			Mixed forest	
		Shrub/Scrub	Young trees	
			Shrub	Native sagebrush
	Herbaceous vegetation and agricultural areas	Grassland/Herbaceous	CRP	Establishment of permanent introduced grasses & legumes
				Establishment of permanent native grasses
			Non‐CRP	
		Agricultural areas	Pasture/Hay	
			Cultivated crops	Oats
				Flax
				Alfalfa
				Barley
				Clover
				Soybean
				Sunflowers
				Spring wheat
				Winter wheat
	Wetland and water	Wetland	Woody wetlands	
			Emergent herbaceous wetlands	Stewart & Kantrud Class II
				Stewart & Kantrud Class III
				Stewart & Kantrud Class IV
		Water	Open water	Stewart & Kantrud Class V
			Snow/Ice	
	Other			

We determined the percentage of studies that used data in each hierarchical level of the classification system in Table 3. The percentage was calculated by dividing the number of times a category was researched by the total number of reviewed studies (26). These were the categories selected by the paper in their method section before they were analyzed, as sometimes the classification changed to better‐fit models during analysis. In cases where a landuse or landcover class was classified differently than in our system, we used our system. For instance, if a paper classified oats as herbaceous (Level 2), we classified them as cultivated crops (Level 5). When the paper thematic classification did not fit any of our system or was missing, it was classified as “other” in our system. This ensured the standardization of the classification across papers. For example, urban environments were classified as other (Level 2).

The spatial grain was the pixel dimensions or stated photograph scale (e.g., 1:3000). If the grain was not stated on the paper, it was classified as unknown to report the occurrence of the missing information. Spatial extent was determined by both explicit reporting (study area) and by estimating it if not clearly stated. This estimation was conducted by assessing study maps and scale bars, considering indicators such as town size, study area, or study block areas, and by summing land cover buffer areas around survey points. If many count routes were present in a state or county (e.g., Breeding Bird Survey routes), the whole state/county was the study area. The percentages of various spatial grains and extents were calculated by dividing the frequency of each grain or extent by the total frequency of remote sensing or land cover products used in the studies. Remote sensing and land cover products were utilized a total of 37 times across the 26 studies. This helped account for the fact that each remote sensing product may have had different grains (depending on the spectral band used and year of remote sensing dataset publication) and extent in each study.

The temporal grain refers to the frequency of remote sensing photography/imagery or land cover mapping use in a study area. The temporal extent denotes the duration of the utilization of the remote sensing product. Such information was linked to the sensor's temporal resolution and the frequency of imagery and land cover updates, tailored to its use in the specific study. For example, until 2024, the NLCD data had a temporal resolution of 3–5 years (now it is yearly) and a temporal extent going from 1992 to 2023 (now it is 1985/86 to present; U.S. Geological Survey 2024a, 2024b). If the study used such data in 2001, 2006, and 2011, the temporal grain would have been 5 years and the extent 10 years. If the temporal grain or extent was unclear, it was classified as unknown to report the occurrence of missing temporal information. The percentages of different temporal grains and extents were calculated similarly to the spatial grain and extent.

The spectral grain determined the habitat type and quality extractable from remote sensing data. Different spectral bands can extrapolate diverse vegetation information that can be relevant to pheasants. For example, natural color photographs (visible spectrum) can only identify broad habitats such as grasslands. Multispectral images using the red and infrared bands can identify vegetation characteristics such as greenness that may be better suited for pheasant habitat identification. The spectral information informs the kind of habitat that can be extrapolated from remote sensing (Gibson and Power 2013a, 2013b; Amirkhiz et al. 2023). The spectral grain was calculated for photographs and images, not landcover or indices (e.g., NDVI), and referred to the number of spectral bands and their width difference in creating the photograph or image. If the spectral grain was not clearly stated in the study, it was estimated from the information given, as indicators of it were often present. For instance, if the study reported that natural color aerial photographs were used, the study most likely used the blue, green, and red bands that respectively range from 400 to 500 nm, 500 to 600 nm, and 600 to 700 nm. In that case, three bands and a spectral resolution width of 100 nm (difference between wavelength ranges) were reported as the most likely scenario. If the spectral grain was not possible to estimate, it was classified as unknown to report the occurrence of such missing information. The percentage of band number and bandwidth difference used was calculated similarly to the spatial grain.

## 3 Results

### 3.1 Temporal Trends

**FIGURE 4:**
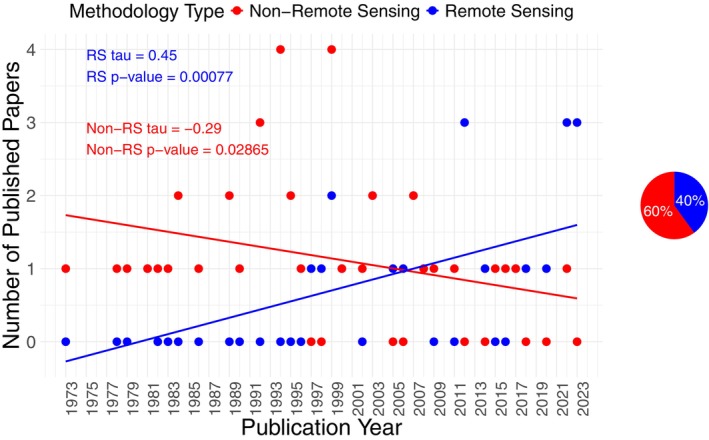
Studies using and not using remote sensing to study pheasant (*Phasianus colchicus*) habitat over time, and their percentage of the total amount of studies found. RS stands for remote sensing.

In the study area the use of remote sensing products in studying pheasant habitat significantly increased over time (tau = 0.45, *z* = 3.36, *p*‐value =0.00077) while non‐remote sensing pheasant studies significantly decreased over time (tau = −0.29, *z* = −2.19, *p*‐value =0.029; Figure 4). Research dates back to 1973 (earliest identified study), with most studies published in the early 1990s. In the 1980s, remote sensing started to be used to study pheasant habitats, becoming more prominent in time. Overall, 26 of the 65 studies (40%) used remote sensing or landcover data (Figure 4).

A total of 19 different remote sensing datasets were utilized in the 26 studies. Four sources had an unknown data collection platform, due to the papers not stating what they were and the information not being readily available elsewhere. All aircraft platforms were manned aircraft (no drones were used) and were used to collect photographs/imagery, except for the USDA Farm Service Agency (FSA) GIS product which had an unknown data type. Similarly, all satellites but one had landcover products derived from them. The satellite from which landcover was not derived provided images from which landcover was derived using unsupervised classification. The greatest percentages among all sources of aircraft‐borne aerial photography/imagery come from private flown aircraft (19%), the National Agriculture Imagery Program (NAIP; 8%) and the Agricultural Stabilization and Conservation Service (ASCS; 8%). NAIP is collected by the United States Department of Agriculture (USDA) at fine spatial resolutions (< 1 m) around every 2 years during the peak agricultural season (U.S. Department of Agriculture 2024). ASCS, which is now part of the Farm Service Agency (FSA), uses remote sensing for land management and agricultural purposes (Farm Service Agency 2016; United States Department of Agriculture 2024). The greatest percentages among all sources of satellite‐based landcover products used in pheasant research were from the National Land Cover Dataset (NLCD; 11%) and the National Wetlands Inventory (NWI; 8%). The NLCD is a Landsat‐based product produced by various federal agencies and produced at irregular intervals between 1992 and 2021, with updates in 1992, 2001, 2006, 2011, 2016, 2019, and 2021, at medium spatial resolution ranging between 30 and 60 m. From 2024 the NLCD started producing landcover annually (U.S. Geological Survey 2024a, 2024b). The NWI is a dataset maintained by the U.S. Fish and Wildlife Service (USFWS), providing wetland locations and information across the United States (U.S. Fish and Wildlife Service 2024; Table 4).

**TABLE 4:** Remote sensing sources and the percentage of times used in the remote sensing ring‐necked pheasant ( *Phasianus colchicus* ) papers.

Agency	Remote sensing source	Percentage of times used	Relevant website
N/A	Aerial photographs	19%	N/A
United States Geological Survey (USGS) and partners	GAP Analysis Program	3%	https://www.usgs.gov/programs/gap‐analysis‐project
	National Aerial Photography program (NAPP)	3%	https://www.usgs.gov/centers/eros/science/usgs‐eros‐archive‐aerial‐photography‐national‐aerial‐photography‐program‐napp
	The National Land Cover Dataset (NLCD)	11%	https://www.usgs.gov/centers/eros/science/national‐land‐cover‐database
	Landsat Thematic Mapper	3%	https://landsat.gsfc.nasa.gov/article/the‐thematic‐mapper/
United States Department of Agriculture (USDA) and partners	Agricultural Stabilization and Conservation Service (ASCS)	8%	https://www.fsa.usda.gov/Internet/FSA_File/vault_holdings2.pdf
	National Agriculture Imagery Program (NAIP)	8%	https://naip‐usdaonline.hub.arcgis.com/
	Cropland Data Layer (CDL)	5%	https://nassgeodata.gmu.edu/CropScape/
	Farm Service Agency's GIS (FSA)	3%	https://www.fsa.usda.gov/Internet/FSA_File/tx_gis_infosheets.pdf#page=3.23
	Veg scape	3%	https://nassgeo.csiss.gmu.edu/VegScape/
	Rangeland Analysis Platform (RAP)	2%	https://rangelands.app/
U.S. Fish and Wildlife Service (USFWS) and partners	National Wetland Inventory (NWI)	8%	https://www.fws.gov/program/national‐wetlands‐inventory
	US Fish and Wildlife Service image data	2%	https://www.fws.gov/office/nebraska‐ecological‐services
U.S. Department of the Interior	General Land Office (GLO)	5%	https://glorecords.blm.gov/default.aspx
Multiple including the following: U.S. Fish and Wildlife Service (USFWS) Nebraska Game and Parks Commission Natural Resources Conservation Service (NRCS) Ducks Unlimited	Rainwater Basin Joint Venture (RWBJV)—Nebraska Landcover	5%	https://www.rwbjv.org/
MicroImages Inc.	GIS micro images	3%	https://www.microimages.com/
Illinois Department of Natural Resources (IDNR)	Illinois Statewide Streams Application layer (SSA)	3%	https://clearinghouse.isgs.illinois.edu/data/hydrology/streams‐and‐shorelines
National Operational Hydrologic Remote Sensing Center	Snow Data Assimilation System (SNODAS)	3%	https://nsidc.org/data/g02158/versions/1#anchor‐data‐access‐tools
N/A	Printed aerial imagery with landcover types	3%	N/A

**FIGURE 5:**
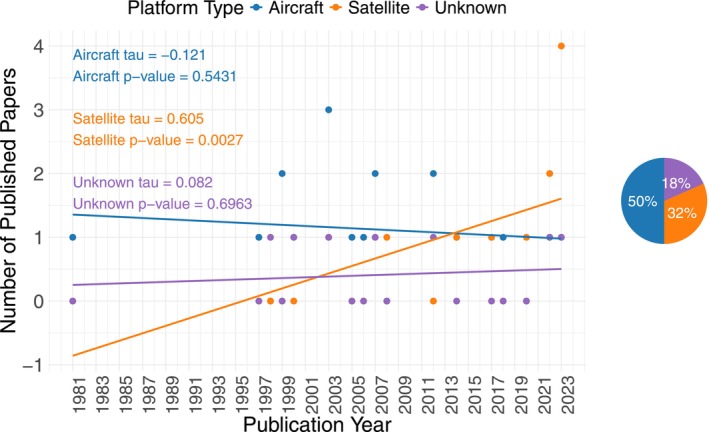
Pheasant (*Phasianus colchicus*) habitat publications depending on remote sensing platforms (i.e., aircraft, satellite, and unknown) over time and their percentage of the total amount of remote sensing studies found.

**FIGURE 6:**
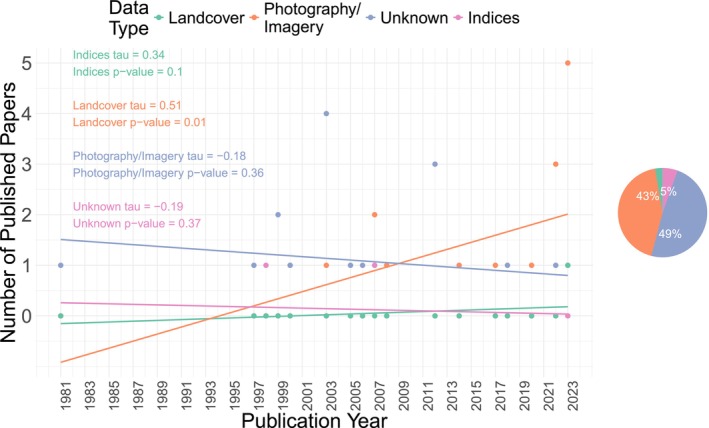
Pheasant (*Phasianus colchicus*) habitat publications depending on remote sensing data type (i.e., indices, landcover, photographs/images, and unknown) over time and their percentage of the total amount of remote sensing studies found.

Remote sensing studies dated back to 1981 with spaceborne products significantly increasing over time (tau = 0.60, *z* = 3.00 *p*‐value = 0.0027) but airborne products exhibiting no temporal trend (tau = −0.12, *z* = −0.61, *p*‐value = 0.54). Eighteen percent of remote sensing products had an unknown platform, due to it not being reported in the study. The explicit report of remote sensing platforms to study pheasant habitat did not change in time (tau = 0.082, *z* = 0.39, *p*‐value 0.70). Overall, airborne products were used the most, followed by spaceborne and unknown platforms. If the unknown category is omitted, 61% of studies used airborne platforms and 39% used spaceborne, in comparison to 50% and 32%, respectively, when it is included. Removing the unknown category does not seem to have a significant effect on the results. Both sets have a higher percentage of aircraft usage rather than satellites, despite the difference between them being slightly higher when the unknown category is removed (Figure 5). The use of landcover products derived from remote sensing significantly increased over time, similar to the use of satellites (tau = 0.51, *z* = 2.56, *p*‐value 0.010), while the use of remote photography/imagery did not change over time (tau = −0.18, *z* = −0.91, *p*‐value = 0.36). Overall, however, the use of remote photography/imagery was slightly greater than that of remote sensing‐derived land cover. Only one study in 2023 researched pheasant habitat using indices (NDVI) and only two studies had unknown remote sensing data type. If the unknown category is omitted, 46% of studies used landcover data, 51% used photography/imagery, and 3% used indices in comparison to 43%, 49%, and 3%, respectively, when it is included. Removing the unknown category does not seem to have a significant effect on the results, as the differences in percentages are minimal (Figure 6).

### 3.2 Thematic Resolution

Most studies that used landcover (88%) focused on cultivated crops in Level 4 of the hierarchical classification system, the “other” category in Level 2 of the classification system (69%), and the Grassland/Herbaceous category in Level 3 of the classification system (54%). All other categories were included less than 42% of the time, with forest and shrub/scrub (Level 2) and herbaceous vegetation and agricultural areas (Level 2) and wetland (Level 3) categories being used the most. A few studies focused on variables in either Levels 1 (coarsest thematic grain) or 5 (finest thematic grain) of the classification system (Table 5).

**TABLE 5:** The thematic grain of ring‐necked pheasant ( *Phasianus colchicus* ) habitat publications.

Level 1	Level 2	Level 3	Level 4	Level 5
Terrestrial and aquatic environments 12%	Forest and shrub/scrub 42%	Forest 31%	Deciduous forest 0%	
			Evergreen forest 0%	
			Mixed forest 0%	
		Shrub/Scrub 4%	Young trees 0%	
			Shrub 8%	Native sagebrush 4%
	Herbaceous vegetation and agricultural areas 42%	Grassland/Herbaceous 54%	CRP 12%	Establishment of permanent introduced grasses & legumes 8%
				Establishment of permanent native grasses 8%
			Non‐CRP 31%	
		Agricultural areas 8%	Pasture/Hay 23%	
			Cultivated Crops 88%	Oats 4%
				Flax 4%
				Alfalfa 12%
				Barley 4%
				Clover 4%
				Soybean 4%
				Sunflowers 4%
				Spring wheat 4%
				Winter wheat 4%
		Wetland 35%	Woody wetlands 0%	
			Emergent herbaceous wetlands 8%	Stewart & Kantrud Class II 4%
				Stewart & Kantrud Class III 8%
				Stewart & Kantrud Class IV 8%
		Water 19%	Open water 15%	Stewart & Kantrud Class V 4%
			Snow/Ice 19%	
	Other 69%			

### 3.3 Spatial Scale

30‐m was the most common spatial grain at 21%, with all but one satellite‐based study using Landsat or Landsat‐derived landcover products. A smaller, but still substantial proportion of studies (11%) used higher resolution data products with grains finer than 4 m. However, grain was not clearly stated for many studies that used airborne photography, leading to a high percentage of studies in the unknown category (43%). Nevertheless, the finer grain tends to come from airborne imagery. If the unknown category is excluded from consideration, the 30 m grain products are even more strongly represented, affirming the dominant use of Landsat or Landsat‐based landcover products. The most common spatial extent was between 100 and 1000 km^2^ (38%; Table 6). These smaller extents were mainly from airborne data, while the larger extents were mostly spaceborne.

**TABLE 6:** Spatial grain and extent used by remote sensing papers researching ring‐necked pheasant ( *Phasianus colchicus* ) habitat.

Spatial scale						
Grain (m^2^)						
1: (6336 and 40,000)	< 4	30	30–90	250	> 100	Unknown
6%	11%	21%	8%	3%	8%	43%
10^a^	19^a^	38^a^	14^a^	5^a^	14^a^	

Extent (km^2^)				
< 100	100 < 1000	1000 < 10,000	10,000 < 1000,000	< 1000,000
14%	38%	16%	27%	5%

^a^ Shows percentages calculated removing the unknown category.

### 3.4 Temporal Scale

The temporal grain of pheasant remote studies was variable, with 35% using the remote sensing product only once, indicating studies' reliance on a static snapshot in time of the habitat rather than long‐term monitoring. Few studies (3%) look at longer‐term environmental changes, namely every 5 or 8 years. Nineteen percent of studies had an annual temporal grain, indicating research focus on annual habitat changes, while a smaller proportion (8%) researched habitat multiple times per year, reflecting an emphasis on seasonal habitat changes. The greatest percentage among all studies (32%) did not clearly report the grain, making it challenging to evaluate with certainty trends in grain sizes over time. When studies with unknown grain are excluded, the results remain similar, with one‐time uses of data remaining dominant. Similarly, the temporal extent of datasets varied, with short‐term studies (lasting less than 5 years) being more common (21%) than long‐term studies (longer than 5 years). The short‐term and long‐term distinction was based on pheasant average lifespan between 1 and 3 years (Baxter and Wolfe 1973; Grahn 1993; Clark et al. 2008). The greatest proportion of studies (35%) had undefined temporal extents due to the use of remote sensing and/or landcover datasets at only one point in time. Thirty‐two percent of studies failed to report the temporal extent—the same proportion of studies that failed to report the temporal grain. When studies with unreported spectral scales are excluded from consideration, results are similar (Table 7).

**TABLE 7:** Temporal grain and extent used by remote sensing papers researching ring‐necked pheasant ( *Phasianus colchicus* ) habitat.

Temporal scale						
Grain						
One time only	Daily	Five times per year	1 year	5 years	8 years	Unknown
35%	3%	5%	19%	3%	3%	32%
52%^a^	4%^a^	8%^a^	28%^a^	4%^a^	4%^a^	

Extent								
N/A	2 years	3 years	4 years	5 years	10 years	11 years	16 years	Unknown
35%	3%	3%	13%	3%	5%	3%	3%	32%
52%^a^	4%^a^	4%^a^	20%^a^	4%^a^	8%^a^	4%^a^	4%^a^	

^a^ Shows percentages calculated removing the unknown category.

### 3.5 Spectral Resolution

Sixteen of the 26 reviewed studies used photographs or imagery, with these being applied 18 times in total, as studies used multiple photographs or imagery sources. Thirty‐nine percent of studies did not report the spectral resolution or spectral width of the photograph/image or stated the source. All papers used multispectral photographs/images, with the most commonly used number of bands being between three and four (44%) and the most common spectral resolutions being moderate and ranging from 100 to 300 nm (34%). A smaller percentage of studies (17%) used from one to three bands with narrower band width, hence higher spectral resolution. When the unknown category is removed, the trend is similar with percentages being more dominant in general (Table 8).

**TABLE 8:** Spectral grain used by remote sensing papers researching ring‐necked pheasant ( *Phasianus colchicus* ) habitat.

Spectral resolution			
Number of bands			
1–3	3	4	Unknown
17%	22%	22%	39%
27%^a^	37%^a^	36%^a^	

	Spectral width (nm)					
50–150	60–140	60–270	100	100–200	100–300	Unknown
11%	5%	5%	6%	17%	17%	39%
18%^a^	9%^a^	9%^a^	9%^a^	28%^a^	27%^a^	

^a^ Shows percentages calculated removing the unknown category.

## 4 Discussion

### 4.1 Temporal Trends

While remote sensing and land cover data are not predominant in pheasant habitat studies in Central North America (40% of studies used remote sensing in total), their use has increased over time. Such dominance in non‐remote sensing products in pheasant habitat research, however, is due to most studies being conducted in the 1990s when remote sensing technology use was not yet prominent (Figure 4). Remote sensing is a cost‐efficient and time‐efficient approach to ecological monitoring that complements field data collection. Fieldwork may provide more accurate fine‐scale habitat estimates than remote sensing but requires a great time and resource investment, especially as the spatial extent increases. Remote sensing has potential to scale up management outcomes in wildlife conservation, especially as technology advances (Campbell and Wynne 2011c; Gibson and Power 2013a, 2013b) and remotely sensed data and computing resources for processing become more accessible. For example, freely available remotely sensed datasets include airborne‐based NAIP and satellite‐based Landsat and Sentinel‐2 imagery (Hansen and Loveland 2012; U.S. Department of Agriculture 2024; European Space Agency 2024) and easily accessible geospatial cloud computing platforms include Google Earth Engine (Gorelick et al. 2017).

Various challenges and opportunities remain in leveraging the full potential of remote sensing and landcover in pheasant research, such as identifying and differentiating important habitat features at fine grains and broad extents (i.e., minimizing scale‐based data tradeoffs). The development of datasets and approaches that link remotely sensed and field‐based datasets to train algorithms capable of identifying pheasant habitat at high spatial resolutions across large areas is a continuing pursuit, especially as new satellite datasets become freely available at finer spatial–temporal resolutions at broad spatial extents. Landcover products are increasing in spatial extent (e.g., WorldCover), and remote sensing products are increasing in their temporal and spatial grain and extent. Furthermore, spectral resolution is increasing with hyperspectral satellites and drones. These technological and data advances increase the potential utility for multi‐scale habitat assessments for pheasants and other wildlife (Cihlar 2000; Campbell and Wynne 2011c; Hansen and Loveland 2012; Gibson and Power 2013a, 2013b; Shahbazi et al. 2014; European Space Agency 2025). Finally, cloud computing platforms such as Google Earth Engine (GEE) support rapid data filtering, processing, and analysis, giving researchers easy access to large datasets that were more challenging to access in the past (Gorelick et al. 2017; Yang et al. 2022; Zhang et al. 2022). At the same time, field data will remain essential for training and validating next‐generation data products and acquiring high‐resolution information that remote sensing cannot; however, remote sensing will likely continue to increase its prominence in ring‐necked pheasant research. Understanding the scale‐based tradeoffs associated with different remote sensing and landcover datasets will enhance research outcomes.

The use of satellite‐derived landcover data have increased in pheasant research over time, particularly landcover derived from Landsat imagery, such as the NLCD. Meanwhile, the use of airborne photographs has remained constant (Figures 4 and 5; Table 4; Campbell and Wynne 2011a). Satellites such as Landsat have been monitoring the Earth for decades at medium‐high spatial and temporal resolution, making it a great source for landcover classification. For example, the NLCD freely provides a comprehensive land cover map of the United States, now at annual temporal resolution (U.S. Geological Survey 2024a, 2024b). Airborne products, on the other hand, cover smaller spatial extents and have coarser temporal grains because of financial and logistical constraints, but can also be used to generate landcover data. There are some freely available airborne datasets that cover broad spatial extents, such as the NAIP; however, the temporal resolution of the data and landcover products derived from it are coarser than that of satellites. These characteristics likely contribute to the popularity of satellite imagery for landcover (Cihlar 2000; Shahbazi et al. 2014; Hansen and Loveland 2012; Grekousis et al. 2015). Overall, however, aircraft photography dominated over satellite‐derived landcover data in remote sensing pheasant studies, probably due to the high proportion of studies with unknown platform sources. The latter were likely satellite‐based, as landcover use was more dominant in pheasant studies and there is an apparent correlation between satellite and landcover use. Additionally, unknown platform sources mainly came from online agencies such as the National Wetlands Inventory database, which likely used freely available satellites but do not explicitly state the remote sensing platforms used in each update (U.S. Fish and Wildlife Service 2024; Figures 4 and 5). It is important to understand why satellite‐derived landcover usage increased with time over other remotely sensed variables, due to the associated remote sensing data sources scales for which pheasant habitat questions can be answered (Hansen and Loveland 2012; Gibson and Power 2013a, 2013b; Shahbazi et al. 2014; Stuart et al. 2019; U.S. Department of Agriculture 2024).

Such an increase in the usage of satellite landcover products is likely because they are becoming more free and widely available and can easily be used and interpreted for large areas. Classification work and associated remote sensing expertise are not required for downloading and using landcover data. The NLCD, National Wetlands Inventory, and CDL were particularly commonly used, likely due to their widespread long‐term availability, standardized classification, and relevance to pheasant habitat (Table 4). Interacting with aircraft‐based platforms and imagery/photography generally requires a higher degree of expertise in remote sensing, which not all pheasant researchers may possess. This is because they must classify the imagery as no equivalent NLCD product is derived from airborne imagery that is publicly available and routinely updated. Satellite landcover can be downloaded and directly used to analyze population trends, while aircraft photography/imagery requires expertise in classifying habitats provided in the remote sensing products before it can be used for analysis. Despite not being used to develop landcover products, the use of airborne photography/imagery remained constant over time, likely due to its finer spatial and spectral resolutions. Airborne imagery was mainly collected by private companies, with governmental products such as the ASCS and NAIP also being important (Table 4; Campbell and Wynne 2011a, 2011b; Hansen and Loveland 2012; Gibson and Power 2013a, 2013b; Shahbazi et al. 2014; Stuart et al. 2019; U.S. Department of Agriculture 2024; U.S. Geological Survey 2024a, 2024b). We encountered no instances of the use of drone imagery in pheasant studies. Drones are becoming widely used in wildlife studies due to minimal habitat disturbance, user friendliness, and fine spectral and spatial resolutions, able to characterize habitat in detail (Mazumdar 2022; Bhatt and Maclean 2023). Planes, however, can uncover broad spatial extents and do not need frequent recharging, unlike drones. Scientists likely did not use drones in their studies due to drones' popularity being recent and most studies being conducted decades ago. Satellite‐derived landcover data will likely remain the primary focus in pheasant habitat studies because of its user‐friendliness, availability, and broad spatial applicability. Satellite technology will advance and offer finer spatial, temporal, and spectral resolutions important to detect pheasant nesting, brood rearing, and wintering habitat. It is important, however, to still recognize the complementary value of other remote sensing platforms, such as aerial photography and UAVs, which may be able to capture targeted pheasant ecological insights that satellites alone may not be able to capture (Campbell and Wynne 2011a, 2011b; Hansen and Loveland 2012; Gibson and Power 2013a, 2013b; Shahbazi et al. 2014; Stuart et al. 2019; U.S. Department of Agriculture 2024; U.S. Geological Survey 2024a, 2024b). Future research should focus on automating habitat classification from satellite, aircraft, and drone imagery/photography so that finer scale pheasant habitat can be identified from remote sensing.

Huge advancements are occurring in image classification that can be tailored toward the assessment of pheasant habitat at multiple scales from various platform sources. The time and labor needed to translate the images into spatial information (i.e., landcover/habitat classifications) is decreasing while opportunities for collaboration between ecologists and remote sensing scientists are increasing (Lang et al. 2009; Mairota et al. 2015; Hu et al. 2021). Geospatial artificial intelligence is now being increasingly used, with machine learning and deep learning, to develop powerful classification algorithms that often out‐compete traditional methods (Rezaee et al. 2018; Himeur et al. 2022; Song et al. 2023). For instance, deep learning technology such as unsupervised learning, multi‐view imaging, and convolutional neural network‐based techniques can increase data organization efficiency to map water delineation in an automated manner (Hu et al. 2021). Furthermore, as technology such as sensors develops, the information gathered from images has higher spatial and spectral resolution. For instance, thermal sensors can be used for wetland mapping (Seiler et al. 2012; Nagendra et al. 2013; Hu et al. 2021). Such advancements are often data‐intensive, and Google Earth Engine is taking the lead in image processing due to its high computational efficiency (Yang et al. 2022; Zhang et al. 2022). Collectively, these advances increase the potential for contributions to pheasant research and management. For example, the new global landcover product “Dynamic World” produces a global landcover dataset at the same spatial and temporal extents and grains as cloud‐free Sentinel imagery. However, its thematic resolution is limited to broad Level 3 categories such as forest, grassland, and cropland, having less detail than Level 4 classifications able to distinguish, for instance, between different grassland types (Brown et al. 2022). Continued advances in classification of satellite, aircraft, and drone imagery will increase opportunities for research to address high‐resolution and multi‐scale habitat questions. Ultimately, the integration of remote sensing and landcover data from satellites, aircraft, drones, and proximal sensors can enable targeted multi‐scale habitat assessments, especially when linked with field data from specific locations and time periods of interest.

### 4.2 Thematic Scale

Pheasant habitat literature predominantly researched cultivated crops (Level 4) and the grassland/herbaceous categories (Level 3), without differentiating among crops or grassland types. Remote sensing in pheasant research rarely investigates habitats at different thematic resolutions despite being able to do so and its importance in impacting results such as pheasant habitat modeling. Thematic resolution can influence the patterns detected in models, the accuracy of validation metrics, the complexity of the analysis, and the interpretation of landscape connectivity (García‐Álvarez et al. 2019; Pelorosso et al. 2021). The thematic resolution relationship with the outcome of the analysis is not always direct, as finer thematic grain does not always equate to more meaningful or accurate results. For instance, finer thematic resolution does not always equate to higher landscape connectivity. Comparing thematic resolution is therefore important in understanding, interpreting, and applying results. Despite spatial resolution tending to have a greater influence on findings than thematic resolution, the latter should still be considered for maximizing the utility of results (García‐Álvarez et al. 2019; Pelorosso et al. 2021). Remote sensing technology is evolving fast and it has already developed enough to investigate different levels of thematic resolution. Below, we explore why different thematic levels may be more researched than others.

Cultivated crops were the most frequently used landuse class in our study area where they also dominate land area (Augustine et al. 2021; Reeves et al. 2021). The focus of many pheasant studies on cropland could be due to its expansion at the expense of other classes, such as grassland and wetland (Li et al. 2019; Reeves et al. 2021; Barg et al. 2025). Cultivated crops are readily identifiable using aerial imagery from aircraft; however, identifying different crop types from aerial imagery can be challenging, especially at the beginning of the growing season when most crops look alike (Campbell and Wynne 2011a; Evett et al. 2020). Many satellite landcover products incorporate cultivated crops in their classification. The NLCD, the Cropland Data Layer (CDL), and the Rainwater Basin Joint Venture Nebraska Landcover identify cultivated crops (Bishop et al. 2011; Rainwater Basin Joint Venture 2012; Wickham et al. 2023; National Agricultural Statistics Service 2024). Only the CDL provides fine enough thematic resolution to differentiate crop types. The CDL, a product derived from Landsat imagery and other data sources such as elevation, soil productivity, and tree canopy, classifies more than 100 types of different crops. Despite this availability, this land cover has only rarely been used in pheasant habitat studies, even though it has been available since 2008 in the conterminous United States (U.S. Geological Survey 2022; National Agricultural Statistics Service 2024). The relative underuse of such products indicates that pheasant research has focused more strongly on cropland generally than on differences among crop types. This highlights an opportunity for future research, as not all crop types may be utilized similarly by pheasants. For example, pheasants in South Dakota generally selected alfalfa, along with small grains and corn during nesting or roosting, with corn usage increasing throughout the progressing season (Hanson and Progulske 1973). Furthermore, pheasants were positively correlated with barley, sugar beets, winter wheat, and sorghum, and negatively correlated with rice and grape crops in California. Such crop usage, however, was affected by multiple factors, such as temperature and crop phenology (Perkins et al. 1997; Coates et al. 2017; Shirley and Janke 2023). Future research may benefit from the use of datasets that differentiate among crop types (e.g., the CDL), in combination with data on additional environmental and habitat factors.

Following cultivated crops, grassland/herbaceous was the next most frequently included landcover class in pheasant studies in our study area, where it also covers a substantial proportion of land area (Augustine et al. 2021; Reeves et al. 2021). Grasslands and herbaceous cover can be distinguished in imagery, although not as easily as cropland (Campbell and Wynne 2011a). Remotely differentiating grassland remains challenging, as they appear spectrally similar in aerial imagery. Many landcover products contain classes for herbaceous/grassland cover, but often their thematic resolution does not distinguish structural classes or successional stages that are ecologically important for pheasants. For example, the NLCD broadly classifies grassland, and the Rangeland Analysis Platform differentiates between annual and perennial forbs and grasses. The Rainwater Basin Joint Venture Nebraska provides a finer classification, such as shortgrass and tallgrass, reflecting more plant associations rather than structural characteristics. The landcover is developed specifically for Nebraska, limiting its broader applicability (Van Andel and Van den Bergh 1987; Warner et al. 2000; Rainwater Basin Joint Venture 2012; Jones et al. 2021; Wickham et al. 2023; Wang et al. 2022). Pheasant studies using remote sensing and landcover only differentiated among pheasant‐relevant grassland types based on supplemental management or field survey data. Pheasants may choose different types of herbaceous fields depending on seasonal needs or habitat structure. For instance, survival was higher in CRPs managed to increase forbs and vertical structure than in unmanaged CRPs and other grasslands in Nebraska (Matthews et al. 2012a). Moving forward, there are opportunities to address questions about differences in habitat structure with finer thematic resolutions in herbaceous landcover classes. Detection of grassland disturbance from remotely sensed imagery might be used as a proxy for structural habitat differences important to pheasants. Depending on intensity and timing, disturbances such as haying, prescribed fire, and grazing can shape grassland structure and composition, making it less or more suitable for pheasants (Hobbs and Huenneke 1992; George et al. 1979; Wang et al. 2022). Development of landcover products that differentiate between various classes of grasslands habitat structure could be advantageous for pheasant research, especially when grassland management data are not easily accessible.

Forest and shrub/scrub with their finer‐level subclasses were not often included in studies, despite their potential importance for pheasants (Berner 1988; Robertson et al. 1993; Taylor et al. 2018; Harsh et al. 2022). In our study area, forests and shrubs are not prominent and can be unfavorable to pheasants at certain spatial extents (Jorgensen et al. 2014; Stuber et al. 2017; Augustine et al. 2021). These habitats house predators detrimental to pheasants; however, not always in a significant manner depending on the woodland type, amount, and juxtaposition. Increased woodland thematic resolution could be used to investigate this aspect further. For example, higher thematic resolution in tree classes could allow for differentiation among different tree and shrub species that may have different responses for pheasants. Expanding such research in North America would increase an understanding of their effect in this continent, as most woodland studies are conducted outside the United States (e.g., Robertson et al. 1988; Draycott et al. 2008; Jorgensen et al. 2014; Neumann et al. 2015; Hall et al. 2021; Chiatante and Meriggi 2022). Most commonly available remote sensing datasets do not contain the spectral resolution required to classify forests and shrubs at a fine‐class level (e.g., NLCD; Rainwater Basin Joint Venture 2012; Wickham et al. 2023; National Agricultural Statistics Service 2024). For more advanced classification at finer levels, light and detection ranging (LiDAR) technology, which provides point clouds that can be used to derive digital surface models that account for aboveground vegetation structure, can be used. This technology, however, is expensive and not freely available across broad extents and at high spatial and temporal resolutions. This means that information on vegetation structure can get outdated quickly due to plant growth, disturbance, etc.…, further limiting the data's utility in pheasant habitat research (Bradbury et al. 2005). Pheasant studies would benefit from the remote study of forest and shrub/scrub; however, biases, technology, and costs need to improve before they are widely researched.

Water‐related environments such as wetlands and snow‐covered zones were also not often included in studies, despite potential importance for pheasants (Berner 1988; Taylor et al. 2018; Harsh et al. 2022). Wetlands, especially in winter, were prominent across many central North American landscapes before their conversion to agriculture (Higgins et al. 2002; Lark et al. 2015; Morefield et al. 2016; Barg et al. 2025). Remote sensing land cover that identifies wetlands and other aquatic environments is available, such as the NLCD or the National Wetlands Inventory. These datasets can go into some detail about the types of wetlands and aquatic environments present; however, they are not often updated, which can be detrimental as they change quickly (Lark et al. 2015; Wickham et al. 2023; U.S. Fish and Wildlife Service 2024). Wetland presence is determined by geology and by temporal fluctuations in precipitation. Geology and weather data, therefore, can also be useful in identifying wetlands (Laubhan and Fredrickson 1997; Tsai et al. 2007; McIntyre et al. 2014). Snow‐covered zones are also important due to associated vegetation and microclimates; however, they are often not researched because most pheasant studies are conducted during the growing season. Some landcover products, such as the NLCD, include perennial but not seasonal snow and ice, while products like Dynamic World detect snow in each Sentinel‐2 image, offering more dynamic insights. Studies should investigate snow depth as it is important for affecting pheasant survival, and it can be assessed using data from The National Snow & Ice Data Center (Covich et al. 1997; Schneider et al. 2011; Homan et al. 2000; NOHRS Center 2015; Tedesco et al. 2015; Brown et al. 2022; Kauth et al. 2022; Wickham et al. 2023; Barg et al. 2025). Such water‐related environments environment complexity, and little prevalence in the landscape may have contributed to the small focus of this environment on pheasants. Future pheasant research could benefit from research conducted in winter with advanced remote sensing datasets with improved thematic, spatial, temporal, and spectral resolution for such environments.

Overall, literature predominantly focuses on grassland and crops, neglecting other classifications and hindering our comprehension of pheasant habitat selection across different thematic resolutions. Land cover classes can be positively or negatively correlated with each other, with implications for statistical modeling. For instance, grasslands and shrubland/woodland tend to be negatively correlated. Such correlation may have impacted the modeling land cover choice in a grassland/cropland dominated landscape by leading the researchers to choose one of the variables to include. Most classification efforts, however, have not included thematic classes relevant to pheasants because many remote sensing data options lack the necessary spectral and temporal resolutions to resolve them. Furthermore, they are primarily based on optical and multispectral image data sets, which are unable to provide detailed identification of habitats. The use of alternative remote sensing options such as hyperspectral and LiDAR offers new possibilities; however, those data lack availability over both space and time, they are not routinely acquired and are expensive (Bradbury et al. 2005; Adam et al. 2010; Campbell and Wynne 2011a; Gibson and Power 2013a, 2013b; Sala and Maestre 2014; Parracciani et al. 2024). LiDAR captures pheasant relevant vegetation structures such as height, unlike multispectral imagery. For example, nesting hens in North Dakota preferred CRP's taller vertical structure (Dubayah and Drake 2000; Rodgers 2002; Genç et al. 2004; Streutker and Glenn 2006; Draycott et al. 2008; Geaumont et al. 2017). Integrating both LiDAR and multispectral/hyperspectral images can improve pheasant habitat classification by combining each methods' strength (Dalponte et al. 2008, 2012; Sankey et al. 2018; Schulte to Bühne and Pettorelli 2018).

Only one study utilized remotely sensed vegetation indices like NDVI to understand pheasant habitat, possibly due to the historical reliance of pheasant research on land cover or less familiarity of pheasant researchers with remote sensing indices. Indices monitor factors such as vegetation dynamics, water bodies, and fire regimes, which are important for pheasants. For instance, fire‐managed CRP was preferred by pheasants in South Dakota (Chiatante and Meriggi 2022; Montero et al. 2023; Orth et al. 2024). Despite such a study finding no significant correlation between NDVI and pheasants, further studies are needed to gauge the potential of remote sensing indices in pheasant research at different spatial and temporal scales.

### 4.3 Spatial Scale

Landsat is the satellite used for most remote sensing land cover products, leading to most pheasant studies having a spatial resolution of 30 m. A 30‐m resolution is suitable for researching pheasant home range and larger spatial extents, as the pheasant home range can vary from around 5 to 300 ha (Whiteside and Guthery 1983; Perkins et al. 1997; Warner et al. 2000). Grassland and ground nesting bird landscape habitat use has been successfully researched by spatial grains of 30 and 250 m from Landsat and MODIS satellites (Bakker et al. 2002; Moreira et al. 2022). Finer spatial grains, however, are needed to research pheasant habitat selection within home ranges (e.g., Matthews et al. 2012a, 2012b). Grains finer than 10 m can be optimal for understanding within home range habitat selection for farmland birds (Sheeren et al. 2014; Li et al. 2022). Only 11% of pheasant studies utilized spatial grains finer than 10 m. Forty‐three percent of studies, however, failed to report the grain, most of which were probably fine since they largely were collected with aircraft platforms, which tend to have fine spatial resolution data (Hansen and Loveland 2012; Shahbazi et al. 2014). Fine spatial resolution (less than 30 m), therefore, is probably prominent in pheasant research. Coarser resolutions were unpopular, with only 3% of studies utilizing them despite their widespread availability, indicating a preference toward assessing specific pheasant habitat within home ranges at a fine thematic grain (Gibson and Power 2013a, 2013b). As finer‐resolution satellite data becomes available, land cover products also become available at finer spatial grains. The European Space Agency (ESA) has developed a few satellites with fine spatial resolution spanning 10–0.65 m, such as Sentinel 2 and SkySat. Sentinel satellites are used to create The Dynamic World land cover from Sentinel‐2 satellites, featuring nine land cover classes at a 10‐m resolution, available every few days at around 75% accuracy. As Sentinel data are freely available, so is the land cover (European Space Agency 2024; Brown et al. 2022; Planet Labs Inc. 2024). The NLCD, in comparison, has an accuracy of 77%–83% (Wickham et al. 2023). Sentinel‐derived land cover datasets were not included in the pheasant studies, likely due to their recent introduction, lesser‐known status, and reduced accuracy compared to the well‐known NLCD. Other satellites with finer resolution are not freely accessible and require a formal application process, limiting accessibility for land cover development (Planet Labs Inc. 2024). As technology advances and prices decrease, remote sensing satellites will have finer spatial resolution, allowing for higher resolution representations of pheasant habitat. Until then, however, aircraft photography/imagery is the best option for fine spatial resolution pheasant habitat research, especially if freely available, as is the case with NAIP imagery. NAIP, however, has relatively coarse spectral and temporal resolutions limiting its application (e.g., Pauly et al. 2018; Shirley and Janke 2023; U.S. Department of Agriculture 2024).

Despite remote sensing being a powerful tool able to survey vast areas of the Earth, most of the remote sensing pheasant studies used spatial extents between 100 and 1000 km^2^, which is one‐third of an average county size in the USA (around 3000 km^2^; U.S. Census Bureau 2014; Table 5). This indicates that existing research is focused at the landscape level across sub‐county extents. When compared to the availability of satellite data across the entire globe and the widespread distribution of pheasants across North America, such extents are small (Hansen and Loveland 2012; BirdLife International 2016). This extent of data use may also have been driven by much data coming from aircraft rather than satellite platforms, with their smaller extents (Gibson and Power 2013a, 2013b; Shahbazi et al. 2014). Evaluating the presence of pheasants across their entire range requires a substantial effort, achievable only through large‐scale surveys such as the Breeding Bird Survey (BBS) conducted across the United States. The spatial extent of pheasant research, therefore, is also limited by the capacity to collect field data on pheasant occurrence. Countrywide bird surveys are valuable for understanding bird population trends, although they may be less precise and focused on pheasants than surveys conducted by experts or biologists in known pheasant habitats. Due to the substantial effort, they also cannot be performed frequently (i.e., at high temporal resolutions; Nielson et al. 2008; United States Department of Agriculture Economic Research Service 2012; Hudson et al. 2017). Only 5% of studies focused on very large extents (> 1000,000 km^2^). More studies (27%), however, focused on statewide and multi‐county extents (10,000–1000,000 km^2^), mostly derived from satellite imagery such as the NLCD. These extents are more feasible to collect field data across. 1000–10,000 km^2^ extents were less common (16% of studies) probably due to their misfit with aircraft or satellite extents. Aircraft is better suited for assessing smaller extents, while satellite is better suited to assess larger ones. Only a minority (14%) of studies focused on small field‐based extents (< 100 km^2^), probably as these extents can be more easily assessed via fieldwork (Gibson and Power 2013a, 2013b). Remote sensing technology is developing to survey animals directly via sub‐meter very high‐spatial resolution. Currently, this technology can use satellites to survey large wild animals, while aircraft can survey smaller animals. However, these methods are expensive, can cause disturbance, and only cover small areas. Such technology has been most successful with big white birds and colonies (BirdLife International 2016; Wang et al. 2019). Until the technology develops to remotely sense pheasants, the extent of remote sensing, especially landcover products, will largely depend on the area used to count pheasants via land surveys.

### 4.4 Temporal Scale

Remote sensing products were often used only once in studies, resulting in the absence of a grain and temporal extent. Such one‐time use could be due to the frequent use of NLCD, which until recently has had a temporal grain of approximately 4 years (beginning in late 2024 the grain has become yearly), more than many studies' time frame (Wickham et al. 2023). In only a few instances, a temporal grain longer than 5 years was reported due to few studies extending longer than this. It could also be that aircraft were often used, and due to their expense, it was more practical to use them once and then rely on field data in the future (Gibson and Power 2013b; Schöttker et al. 2023). Researchers may have also not considered looking at habitat selection at multiple temporal scales (Wheatley and Johnson 2009) and this is highlighted by the fact that many studies failed to report the temporal grain and extent. Not reporting the temporal scale indicates a documentation gap and a lack of focus on the temporal grain and extent of the study. Having just one snapshot in time of the environment limits the understanding of pheasant habitat relationships, as the landscape changes quickly, and pheasant habitat choice differs seasonally (Barg et al. 2025). Future research should use remote sensing to explore pheasant habitat at multiple temporal scales.

When the remote sensing product was used repeatedly, it was mostly used annually for a short temporal extent. This is likely because many remote sensing landcover products are updated yearly (e.g., CDL and The Rangeland Analysis Platform). Such annual updates usually come during the growing season and still represent one yearly snapshot of a fast‐changing landscape and pheasant habitat decision (Jones et al. 2021; U.S. Department of Agriculture 2024; National Agricultural Statistics Service 2024). Amirkhiz et al. (2023) used Landsat and MODIS products available multiple times within the year to overcome the limitations of annual landcovers. For example, the Landsat‐based Rangeland Analysis Platform (RAP) describes vegetation productivity every 16 days, and MODIS products offer biweekly land surface temperature data, both important aspects for pheasant habitat. Amirkhiz et al. (2023) is the most recent study of the ones summarized by this review and provides a valuable model for future research. Furthermore, free remote sensing data span decades, so the lack of long‐term studies using remote sensing may be due to short project timelines due to research focus rather than the technology (Bakker et al. 2010; Gibson and Power 2013a, 2013b). Broad temporal extents, however, are important to understand pheasant habitat with environmental change (Lindenmayer et al. 2012). It would be beneficial to have access to free or inexpensive landcover data that can be used multiple times throughout a study period, ideally several times within a year, such as the Dynamic Word landcover albeit at finer thematic grain (Brown et al. 2022). A study duration of at least 5 years (depending on the study objectives) is recommended to align with the pheasant's lifespan (between 1 and 3 years), to capture significant multigenerational long‐term ecological trends (Baxter and Wolfe 1973; Grahn 1993; Clark et al. 2008). This would greatly aid in assessing pheasant habitat at various time scales relevant to pheasants.

### 4.5 Spectral Scale

Pheasant remote sensing literature did not utilize hyperspectral data, which has hundreds of narrow spectral bands, to classify pheasant habitat at fine thematic grain. Multispectral data, which have few broad spectral bands, were preferred to broadly classify landcover at coarse thematic grains. Hyperspectral data are not as widely available as multispectral data and require expertise or tools to manage it due to high computational resources and memory use. Multispectral data are more manageable and therefore preferred by pheasant researchers (Christophe 2011; Bioucas‐Dias et al. 2013; Adão et al. 2017). Such results align with the thematic grain results which are coarse and focused on general grassland and cropland from publicly available Landsat‐based datasets. Some pheasant studies used narrower band sensors, which are better suited for specific habitat assessment; however, they did not qualify as hyperspectral data. Multispectral remote sensing data covering the visible and occasionally the near‐infrared bands with broad 100 nm width bands were often used in pheasant studies and are well suited for general habitat characterization. Landsat imagery products freely provide such data for large‐scale habitat assessments. A smaller percentage of studies relied on 1–3 bands from aerial images, providing information for manual landcover classification. Such studies may have relied on field validation rather than spectral analysis to assess pheasant habitat. Almost half of the studies did not report spectral resolution or spectral width. Such studies come from aircraft sensors before 2012, so they likely relied on multispectral data with coarse spectral grain (Marcus 2002; Legleiter 2003; Campbell and Wynne 2011b; Bradter et al. 2020). These studies likely did not rely on spectral information for habitat assessment but rather on spatial resolution, outlining the need for pheasant researchers to better understand spectral data to improve habitat assessments. As more pheasant studies uncover habitats at fine thematic grain, it is expected that more hyperspectral data with finer spectral grain (more bands and narrower widths) will be used to understand pheasant habitat. To be applied successfully, such use needs to come with better understanding of spectral data in remote sensing.

## 5 Conclusion and Implications

The findings of this review are useful for guiding future research by identifying the most commonly used remote sensing technologies and datasets, those that are underutilized, and additional tools that could enhance pheasant habitat research. Pheasant remote sensing studies are increasingly relying on satellite landcover products and less on aircraft photography/imagery, likely due to their increased availability and user‐friendliness, largely focusing on cultivated crops and grassland/herbaceous classes. Furthermore, they focus on medium‐fine spatial grains due to being derived from Landsat, which is often the remote sensing tool of choice but not widely used across large extents, likely due to the limitations of surveying pheasants. Remote sensing products are also often only used once, highlighting the lack of focus on researching pheasant habitat at multiple temporal scales. Finally, pheasant studies do not use hyperspectral data, focusing on getting habitat information from a few bands with large wavelength widths. These insights will help researchers make informed decisions when selecting appropriate remote sensing data and methodologies for future investigations. Remote sensing is increasingly being used in pheasant studies, and with its advancement, will likely become more relevant and more precise in assessing pheasant habitat at different scales.

The reliance on the aforementioned scales can limit the effectiveness of conservation efforts, as they mainly explore coarse‐scale patterns rather than finer ones. Currently, management efforts are informed by broad habitat assessments which may overlook the finer scales required for pheasant survival. For example, while pheasants may broadly select grassland, they may specifically rely on habitat mosaics of grassland at different post‐disturbance successional stages. Research that focuses only on broad grassland classifications, without accounting for finer thematic grain, may miss crucial pheasant habitat information, potentially misinforming management practices. As a result, conservation resources may be allocated to establish broad grasslands that do not entirely meet pheasant needs. Furthermore, single‐time point habitat assessments cannot describe seasonal shifts in pheasant habitat use. Management decisions based on static landscape snapshots do not consider dynamic factors such as disturbances (e.g., habitat loss from agriculture or fire) or weather events that influence pheasant habitat selection throughout the year. This contributes to a limited understanding of pheasant habitat requirements over time and the limited effectiveness of conservation efforts (Gabbert et al. 1999; Bissonette 2012; Ronnenberg et al. 2016; Porter et al. 2021; Orth et al. 2024). Tools such as Sen2Chain and GEE_xtract offer promising solutions by processing large satellite time series, thereby simplifying environmental information in time and space. These tools support a deeper understanding of temporal and spatial trends in pheasant habitat selection. Future research should utilize such tools to better explore pheasant habitat use at multiple temporal and spatial scales, for more informed and adaptive conservation strategies (Révillion et al. 2024; Valerio et al. 2024).

Several studies failed to clearly report their spatial, temporal, and spectral scales, limiting our understanding of what scales were truly used in the studies. This should be taken into consideration when interpreting results, as more scales may have been studied but were unreported. As habitat findings are restricted by the scale, however, such studies did not greatly contribute to the greater pheasant habitat knowledge (Johnson 1980; Godvik et al. 2009; Mayor et al. 2009). Furthermore, this study did not consider remote sensing products such as land surface temperature or soil moisture, which can be important factors for pheasant habitat selection. Future research could benefit from exploring these factors as well (Francis 1968; Gabbert et al. 1999; Homan et al. 2000). Remote sensing is only used to research a part of pheasant habitat, and a more comprehensive habitat research at different scales than the ones described would greatly benefit pheasant conservation. Remote sensing can be a powerful tool for researching pheasant habitat at multiple scales, and now that a better understanding of its bias is present, research can focus on uncovering ignored scales.

The best remote sensing technology for assessing pheasant habitat should be selected based on the scale and research question to address biases and knowledge gaps in research. Table 9 provides a list of freely available remote sensing products that will help research such gaps with their suitability to research pheasant habitat. In our review, the NLCD was the most popular satellite land cover, applied for multiple broad scale assessments. It has been utilized to assess pheasant occupancy in Illinois, nesting survival in South Dakota CRP fields, the effects of land cover change in Kansas, and the relationship between CRP and pheasant populations nationwide, consistently showing that grassland has beneficial effects on pheasants (Nielson et al. 2008; Schindler et al. 2020; Solem and Runia 2022; Emmet et al. 2023). The NAIP was the most frequently used freely available aerial imagery source. It was used to investigate fall‐seeded crops in Iowa, finding little evidence of their benefit for pheasant cover. In South Dakota, it supported studies on CRP nesting survival, confirming their value, and on pheasant productivity, revealing positive relationships with CRP and cereal grains (Pauly et al. 2018; Solem and Runia 2022; Shirley and Janke 2023). While the NLCD was used for broad scale assessments, the NAIP supported finer ones, both sources highlighting the importance of grasslands for pheasants. Future research can benefit from both datasets to address pheasant habitat questions at multiple spatial scales.

**TABLE 9:** Useful remote sensing datasets to classify ring‐necked pheasant ( *Phasianus colchicus* ) habitat at different scales.

Remote sensing dataset	Thematic grain	Spatial scale		Temporal scale		Spectral grain	Suitability for pheasant habitat (Barg et al. 2025)
		Grain	Extent	Grain	Extent		
**National Land Cover Dataset (NLCD)** U.S. Geological Survey (2024a); U.S. Geological Survey (2024b)	Level 1–3 and most Level 4	30 m (Landsat‐based)	Conterminous United States	Originally around 4 years, now (2024) annual	From 1985 to present	N/A	Useful for broad scale pheasant habitat assessment
**Cropland Data Layer (CDL)** National Agricultural Statistics Service (2024)	Cultivated crops (Level 5)	30 m (Landsat‐based)	Conterminous United States (earlier years just included a few states)	Annual	From 1997 to present	N/A	Suitable for identifying different crops that are important for pheasants (e.g., corn and soybean), at medium‐fine spatial and temporal scales
**Global Ecosystem Dynamics Investigation (GEDI)** NASA (2024)	Forests/shrubs (Levels 4–5)	25 m	Global	Daily	From 2018 to present	N/A	Best for forest/shrub characterization such as forest canopy and tree height at fine spatial and temporal grain. Relevant for assessing pheasant cover and roosting locations
**The National Wetland Inventory (NWI)** U.S. Fish and Wildlife Service (2024)	Wetlands (Levels 1–4)	Variable from around 1:80 K to 1:40 K	Conterminous United States, Hawaii, Puerto Rico, the Virgin Islands, Guam, the major Northern Mariana Islands and Alaska	Twice a year	From 1979 to present	N/A	Suitable for broad wetland classifications at medium‐fine spatial and temporal scales. Relevant to wintering pheasant habitat
**National Agriculture Imagery Program (NAIP)** U.S. Department of Agriculture. (2024)	Potential for Level 5 classification	Less than 1 m	Most of the United States	Annual	From 2002 to present	Four‐band imagery (RGBNIR)	Potential for fine thematic habitat characterization at fine spatial and temporal scale and medium spectral scale; Expertise is required for classification. Potentially relevant for pheasants at all seasons
**World Cover** European Space Agency (2025)	Most Levels 3 and 4	10 m	Global	Annual	From 2020 to 2021	N/A	Useful for broad scale pheasant habitat assessment at a finer spatial grain than NLCD
**Dynamic World** Brown et al. (2022)	Most Levels 3 and 4	10 m	Global	Near real time	2015 to present	N/A	Useful for broad thematic scale pheasant habitat assessment at very fine temporal grain
**Rangeland Analysis Platform** McCord et al. (2023)	Herbaceous (annual and perennial forbs and grasses; Level 3/4)	30 m	Conterminous United States	16 days	1986 to present		Useful to assess seasonal grassland pheasant use at medium spatial grain

Emerging and integrative remote sensing datasets provide opportunities to enhance pheasant habitat research across spatial and temporal scales. Products such as the ESA World Cover and Dynamic World, though not yet used in pheasant studies, hold promise due to their finer spatial resolution (10 m) compared to commonly used datasets like the NLCD and CDL. Their thematic resolution, however, is coarse, which may limit their application for fine thematic habitat assessments. Dynamic World also offers almost real‐time temporal grain, being able to track pheasant habitat selection at almost real‐time (Brown et al. 2022; The European Space Agency 2025). Furthermore, RAP is another promising tool and dataset due to its specificity for grassland and high temporal grain of 16 days for its herbaceous biomass product. RAP could be suited for assessing nesting and brood rearing habitat; however, it would be more useful if it had a finer thematic grain describing composition and structure. These are important features for pheasants during those stages of life (Riley et al. 1998; Clark et al. 1999; McCord et al. 2023). This is especially important considering that RAP has higher percent cover and biomass estimates error in the Great Plains (around 10% error; Harrison et al. 2025). The suitability and accuracy of remote sensing datasets for addressing various pheasant research questions can be enhanced by integrating multiple data sources. For example, SPOT 5 satellite imagery combined with phytosociological data enabled the creation of vegetation maps able to detect grassland bird habitat preference along moisture and vegetation gradients. Similarly, LiDAR and multispectral fusion data were the best predictor of stem density, important for the detection of songbirds in multiple years (Gallant 2009; Swatantran et al. 2012; Besnard et al. 2015). Future pheasant research should adopt such integrative approaches to answer pheasant habitat relationship questions at the desired scales.

Future advancements in remote sensing should prioritize the development of products with higher temporal resolution, at least bi‐monthly, depending on the season to better capture habitat dynamics during nesting, breeding, foraging, and wintering cycles. The NLCD, which historically had a 4‐year update cycle, has recently shifted to annual temporal resolution. Continued efforts toward more frequent updates, possibly seasonal or bi‐annual products, would significantly enhance pheasant habitat monitoring capabilities. Furthermore, improved grassland classification capturing structure and composition would be crucial for identifying nesting and brood‐rearing habitat. Current land cover datasets often group grasslands into broad categories, failing to distinguish critical variations in vegetation height, density, and composition that affect pheasant habitat suitability. LiDAR and hyperspectral technology offer promising solutions for enhancing grassland classification. Winter habitat monitoring is another gap in remote sensing pheasant habitat research, as most remote sensing products are limited to the growing season. This seasonal limitation can overlook critical winter habitat components, such as residual vegetation cover, thermal wetlands, and woodlands refuge, and the availability of food resources post crop harvest that can be essential for pheasant winter survival. High‐resolution airborne photographs have been successfully used to estimate winter vegetation cover for grassland birds, and spaceborne imagery can be used similarly (Table 9; Riley et al. 1998; Clark et al. 1999; Rodgers 2002; Leif 2005; U.S. Geological Survey 2019; Montes‐Aldaba et al. 2018; Amirkhiz et al. 2023; Barg et al. 2025).

Future studies should focus on using products with finer/different thematic grains other than general grasslands and crops outlined in Table 4, large spatial extents, and temporal grain that represents habitat multiple times within each year. This will help further align the use of remote sensing and landcover in pheasant research with pheasant ecology and habitat. Studies should also clearly report the scale they are using when assessing pheasant habitat, as this information was often unclear and hard to gather. Furthermore, future research would benefit from comparing remote sensing applications for pheasant habitat at different scales with other grassland species. Examining how fine resolution datasets have improved habitat insights for these species could enhance methodological applications and enhance the potential of remote sensing tools for pheasant conservation. This study is fundamental for pheasant conservation, as it can help guide future research to improve our understanding of pheasant habitat with a technology that is likely to become increasingly more relevant in wildlife studies. Such understanding will inform pheasant management, aiding conservation in the Great Plains and beyond (Kushwaha and Roy 2002; McDermid et al. 2009; Vogeler and Cohen 2016).

## Author Contributions

**Megan Baldissara:** conceptualization (equal), data curation (equal), formal analysis (lead), investigation (lead), methodology (equal), visualization (lead), writing – original draft (lead). **Allison Barg:** conceptualization (equal), data curation (equal), methodology (equal). **Andrew Little:** conceptualization (equal), funding acquisition (lead), methodology (equal), supervision (supporting), writing – review and editing (equal). **Zhenghong Tang:** conceptualization (supporting), writing – review and editing (equal). **Brian Wardlow:** conceptualization (supporting), writing – review and editing (equal). **Daniel R. Uden:** conceptualization (equal), methodology (equal), supervision (lead), writing – review and editing (lead).

## Conflicts of Interest

The authors declare no conflicts of interest.

## Data Availability

The data used to support the findings of the study are openly available in “Dryad” at https://doi.org/10.5061/dryad.xsj3tx9rb.
